# Cancer-associated adipocytes: emerging supporters in breast cancer

**DOI:** 10.1186/s13046-020-01666-z

**Published:** 2020-08-12

**Authors:** Chongru Zhao, Min Wu, Ning Zeng, Mingchen Xiong, Weijie Hu, Wenchang Lv, Yi Yi, Qi Zhang, Yiping Wu

**Affiliations:** Department of Plastic Surgery, Tongji Hospital, Tongji Medical College, Huazhong University of Science and Technology, 1095 Jiefang Avenue, Wuhan, 430030 Hubei China

**Keywords:** Breast cancer, Adipocytes, Cancer-associated adipocytes, Adipokines, Breast cancer therapy

## Abstract

Breast cancer (BC) is a malignant breast tumor confronted with high invasion, metastasis and recurrence rate, and adipocytes are the largest components in breast tissue. The aberrant adipocytes, especially the BC-neighbored cancer-associated adipocytes (CAAs), are found in the invasive front of BC. CAAs present a vicious phenotype compared with mature mammary adipocytes and mediate the crosstalk network between adipocytes and BC cells. By releasing multiple adipokines such as leptin, adiponectin, interleukin (IL)-6, chemokine ligand 2 (CCL2) and chemokine ligand 5 (CCL5), CAAs play essential roles in favor of proliferation, angiogenesis, dissemination, invasion and metastasis of BC. This article reviews the recent existing CAAs studies on the functions and mechanisms of adipocytes in the development of BC, including adipokine regulating, metabolic reprogramming, extracellular matrix (ECM) remodeling, microRNAs (miRNAs) and immune cell adjusting. Besides, adipocyte secretome and cellular interactions are implicated in the intervention to BC therapy and autologous fat grafting of breast reconstruction. Therefore, the potential functions and mechanisms of CAAs are very important for unveiling BC oncogenesis and progress. Deciphering the complex network between CAAs and BC is critical for designing therapeutic strategies and achieving the maximum therapeutic effects of BC.

## Background

Breast cancer (BC) is the most frequently diagnosed malignancy and the major cause of cancer-related death in females worldwide [1]. The reason is that BC is confronted with high invasion, metastasis and recurrence rate, especially the metastatic capacity to the brain and lungs [2]. Adipose tissue plays a crucial role as an energy storage depot and can act as endocrine cells to produce various bioactive substances [3]. Epidemiological and etiological evidence has shown that the aberrant adipose state such as obesity is a significant risk and negative prognosis factor for BC [4]. Besides, mounting studies have confirmed that adipocytes adjacent to invasive cancer cells, which are referred to as cancer-associated adipocytes (CAAs), are involved in the progression of BC [5]. Thus, the aberrant adipose tissue, especially the BC-neighbored CAAs, is a BC hallmark which has been confirmed in human BC samples [6].

The breast is a unique and dynamic organ that changes in anatomy and function continuously throughout the whole life. Based on the anatomical distribution, a fully differentiated breast is composed of two compartments: the epithelial compartment that consists of glands with branched ducts and lobuloalveolar differentiated units; and the connective tissue compartment, commonly known as the breast fat pad, is primarily constituted by adipose tissue [7]. Normally, mature adipocytes and epithelial cells are separated by the basement membrane, limiting the possibility of interaction between the two cells. However, BC cells will directly expose to the tumor microenvironment (TME) containing adipocytes when they break through the basement membrane [8]. As a major cellular component of the breast tissue stroma, adipocytes are involved in all BC processes, consequently promoting cancer progression. However, the highly complex interaction orchestrated by CAAs and BC cells has not been completely elucidated.

**Fig. 1:**
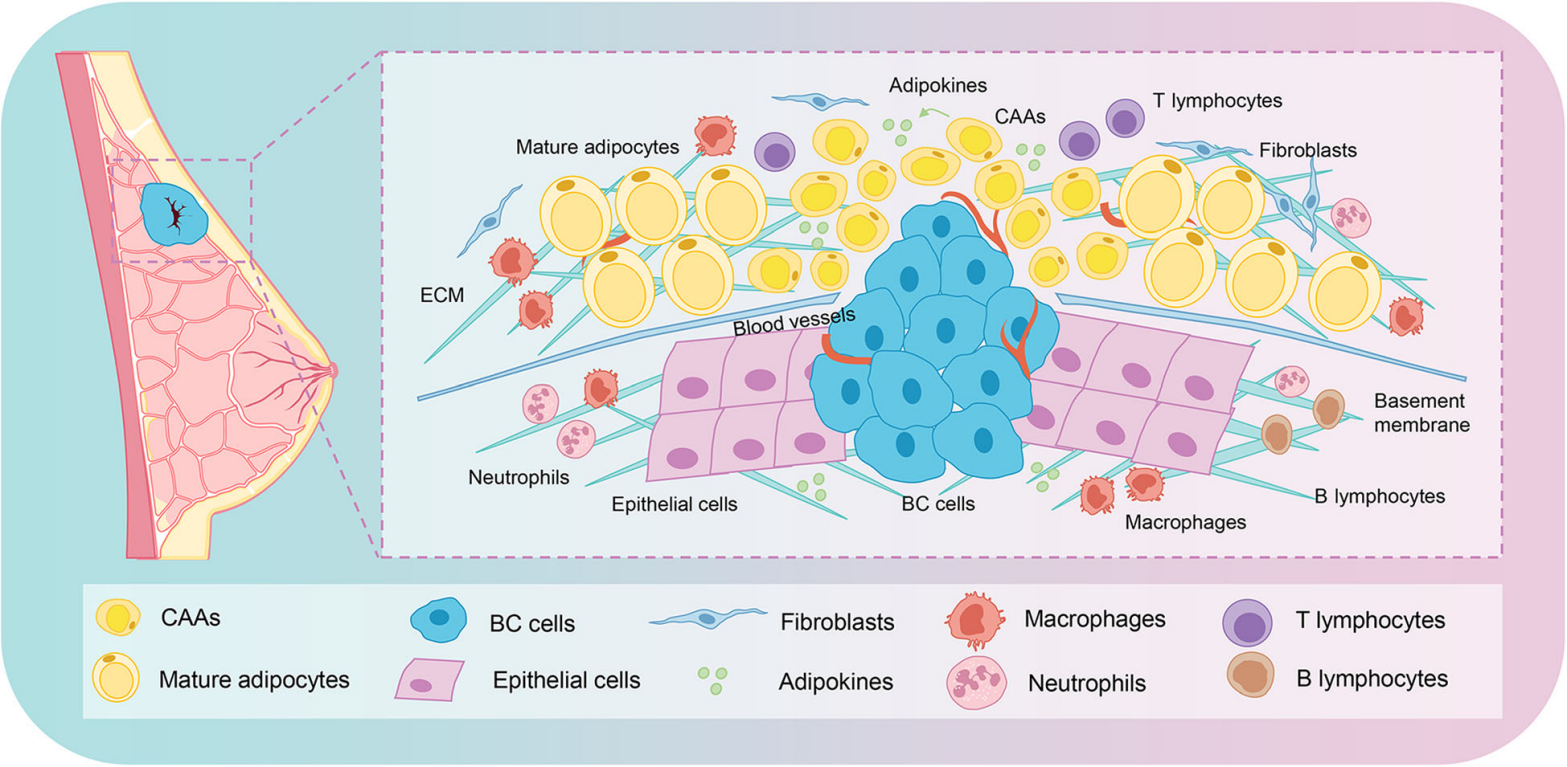
Adipocytes change into CAAs in the invasive front of breast cancer. Normally, mature adipocytes and epithelial cells are separated by the basement membrane, limiting the possibility of interaction between both cell types. However, BC cells will directly expose to the TME containing adipocytes when BC cells break through the basement membrane. The adipocytes adjacent to the BC cells then change into CAAs, which are characterized by reduced volume, a gain of an irregular shape with smaller and dispersed lipid droplets, and have an important role in promoting progression and metastasis of BC. Abbreviations: BC, breast cancer; TME, tumor microenvironment; CAAs, cancer-associated adipocytes; ECM, extracellular matrix

The interplay between the neighboring CAAs and BC ultimately results in shaping the TME towards the oncogenic-driven state in favor of proliferation, angiogenesis, tumor dissemination, invasion and metastasis (Fig. 1). This article has reviewed the recent existing studies on the mechanisms of adipocytes in the development of BC, including adipokines regulating, metabolic reprogramming, extracellular matrix (ECM) remodeling, microRNAs (miRNAs) and immune cells adjusting. It also emphasized the clinical implication of the resistant effect of adipocytes on BC therapy, and the potential effect of autologous fat grafting after mastectomy on BC recurrence. An in-depth understanding of the mechanisms of adipocytes in BC development will provide better insight into CAA-associated tumorigenesis and screen novel strategies for therapeutic interventions of BC.

## Adipocytes and breast cancer

Adipose tissue plays an important physiological role as a metabolically active storage compartment and endocrine organ due to its diverse abilities in secreting various adipokines [9]. It has aroused great interest in understanding the development and renewal of adipose tissue under normal or pathological conditions, such as obesity and BC. Adipocytes constitute the main cell component of ECM in BC [10, 11]. CAAs are not only found adjacent to BC cells but also communicate with BC cells by releasing various factors that can mediate local and systemic effects. Adipose tissue dysfunction has been linked to accelerated growth and survival of BC cells. Numerous studies have confirmed that CAA-BC cell crosstalk can promote BC progression and metastasis through multiple secreted adipokines, and their role in remodeling tumor characteristics.

### Mature adipocytes vs. cancer-associated adipocytes

Physiologically, adipocytes that constitute breast tissue and encapsulate around the mammary gland, can maintain normal breast morphology and the energy balance. These mature adipocytes are mainly white adipocytes (WAT), with round shape, large unilocular lipid droplets and endocrine functions [12, 13]. CAAs mainly locate at the invasive front of breast tumors, namely adjacent to BC cells. Notably, compared with mature adipocytes, CAAs possess smaller cell sizes, irregular shapes, and small/dispersed lipid droplets [14, 15]. Moreover, CAAs exhibit a modified phenotype characterized by the loss of lipid content, decrease in late adipocyte differentiation markers and overexpression of inflammatory cytokines and proteases [14]. In terms of adipokines, secretions of leptin, adiponectin, interleukin (IL)-6, chemokine ligand 2 (CCL2), chemokine ligand 5 (CCL5) and other cytokines in CAAs are more aberrant than that in mature adipocytes [5, 16]. Most importantly, compared with the distant mature adipocytes, CAAs could gain a more vicious phenotype, and enhance BC growth and metastasis, by a less physical barrier and more aggressive secretome of adipokines (as described in Table 1).

**Table 1:** Mature adipocytes vs. cancer-associated adipocytes

	Mature adipocytes	CAAs
Definition	Adipose tissue occupies 56% of the non-lactating breast tissue and 35% of lactating breast tissue. Adipocytes that constitute breast tissue are WAT, with lipid-storage and endocrine functions.	Adipocytes at the invasive front of breast tumor exhibit a modified phenotype, a loss of lipid content, a decrease in late adipocyte differentiation markers and overexpression of inflammatory cytokines and proteases.
Location	Mature adipocytes encapsulate around the mammary gland; Adipocytes are separated with epithelial cells by the basement membrane.	Locate at the invasive front of breast tumor; Adjacent to BC cells.
Morphology	Normal cell size; Round shape; Large unilocular lipid droplets.	Smaller cell size; Irregular shape with smaller size; Smaller and dispersed lipid droplets.
Function	Maintain normal breast morphology; With endocrine functions and maintain the energy balance; Normally expression of adipokines, including leptin, adiponectin, IL-6, CCL2, CCL5 and other cytokines.	Aberrantly expressional secretion of adipokines, including leptin, adiponectin, IL-6, CCL2, CCL5 and other cytokines.
Impact on breast cancer	The promoting effect of adipocytes on BC is far less compared with CAAs; The risk of BC might increase under the condition of obesity.	The interaction between CAAs and BC cells may directly affect their morphology and function; CAAs could promote BC cell proliferation, viability, migration and invasion in vitro, and could enhance tumor growth and metastasis in vivo xenograft studies in a paracrine manner.

Abbreviations: *WAT*, white adipocytes; *BC*, breast cancer; *IL-6*, interleukin 6; *CCL2*, chemokine ligand 2; *CCL5*, chemokine ligand 5; *CAAs*, cancer-associated adipocytes

### Adipokines

Adipocytes can secrete more than 600 kinds of metabolites, hormones and cytokines, collectively known as adipokines. These adipokines are involved in the regulation of multiple life activities, including fat distribution, insulin secretion, energy consumption, inflammatory reaction [5]. Adipokine-mediated crosstalk network between adipocytes and BC cells favors the proliferation, survival and metastasis of BC [17]. Here, we especially focus on the emerging roles of CAA-derived leptin, adiponectin, IL-6, CCL2, CCL5 and other key adipokines in this reciprocal crosstalk. Uncovering the functions and the mechanisms of CAA-derived adipokines are essential to understand the BC behavior and the establishment of accurate interventions.

#### Leptin

Leptin, a hormone with a molecular weight of about 16 kDa, is encoded by the LEP gene on human chromosome 7 and is mainly synthesized and secreted by adipocytes. By binding to its receptor ObR, leptin exerts diverse biological functions including proliferation, differentiation, inflammation and nutrient absorption [18]. Leptin is correlated with BC occurrence and tumor behavior. There is a positive correlation between the elevated serum leptin level and BC risk, especially in obese or postmenopausal women [19, 20]. Furthermore, the leptin expression was increased in adipose tissue in the invasive front compared with that in the distant BC site [21]. In addition, the leptin production in CAAs was increased compared with mature adipocytes, suggesting that leptin was involved in the interaction of adipocytes and BC cells [13]. Leptin may modulate many aspects of breast tumorigenesis from initiation, growth to metastatic progression by autocrine, endocrine and paracrine manner.

Many studies have advocated the importance of leptin in promoting BC growth [5, 13]. In addition, the crucial role of leptin in the invasion and metastasis-associated events of BC is also in focus. The results from Wei et al. showed that leptin could promote epithelial-mesenchymal transition (EMT) in BC cell lines via up-regulating the expression of pyruvate kinase M2 (PKM2) and activating the PI3K/AKT signaling pathway in vitro and in vivo [22]. Using MCF-7 and MDA-MB-231 cell lines, Juárez-Cruz also confirmed that leptin could enhance secretion of ECM remodelers by activating focal adhesion kinase (FAK) through an Src- and STAT3-dependent canonical pathway, suggesting that leptin was associated with the establishment of a more aggressive invasive phenotype in BC cells [23]. When BC cell lines cocultured with mature adipocytes, adipocyte-derived leptin and IL-6 promoted local invasion and eventually metastasis of tumor cells via activation of lysyl hydroxylase 2 (PLOD2) [24]. Moreover, adipocyte-derived leptin was approved to propel self-renewal of BC stem cells and chemoresistance via activating JAK/STAT3-regulated fatty acid β-oxidation (FAO) in mouse breast tumors in vivo [25]. Local adipocytes enable BC progression via leptin-regulated immune cells. Li et al. investigated that leptin triggered macrophage-related cytokine IL-18 production via NF-κB/NF-κB1 signaling in tumor-associated macrophages (TAMs), while via PI3K-AKT/ATF-2 signaling in BC cells, possibly contributing to BC invasion and metastasis [26]. Leptin enriched in mammary adipocytes could downregulate CD8^+^ T cell effector functions through activating STAT3-FAO and inhibiting glycolysis, leading to inhibition of breast tumor development [27].

#### Adiponectin

Adiponectin, a 30 kDa peptide hormone encoded by the AdipoQ gene, plays a major role in glucose metabolism and energy homeostasis. Adiponectin has been confirmed to be associated with diabetes, inflammation and cancer development [28]. Adiponectin act mainly through two adiponectin receptor subtypes, AdipoR1 and AdipoR2, which are identified in mammary tissues obtained from both humans and animals [29]. However, the role of adiponectin receptors in BC development remains to be established. Tuna et al. measured expression levels of adiponectin, AdipoR1 and AdipoR2 proteins in the mammary fat pad, mammary tumor (MT) and serum adiponectin levels from 74 weeks old mice with and without MT [29]. The results showed that protein expression levels of adiponectin and AdipoR1 were significantly lower in MTs compared to control tissues, revealing that AdipoR1, rather than AdipoR2, might be dominant in adiponectin signaling in MT development [29]. Further studies are still necessary to characterize the roles of these two receptors in tumor development. Notably, reports have shown that adiponectin levels in adipose tissue of BC were significantly lower than that of normal breast adipose tissue, while the adiponectin receptor mRNA expression levels were significantly increased [6]. As the secretion of adiponectin was decreased in CAAs, it implied the potential relationship between adiponectin and its related signaling proteins in anti-BC activities.

Adiponectin could negatively regulate cancer cell growth via regulating inflammatory signaling molecules, including Erk1/2, Akt, tumor necrosis factor (TNF)-α, IL-1β, NF-κB, IL-6, IL-8 and CCL2 [20]. Autophagy is a basic vacuolar, lysosomal degradation process known to help to prevent the accumulation of damaged proteins and organelles, recycling cytoplasmic constituents, and maintaining cellular homeostasis [30]. CAAs are of functional diversity and may enhance malignant behavior by inducing the senescence of adipocytes via autophagy, such as transforming growth factor (TGF)-β, adiponectin, or hypoxia-inducible factor (HIF)-1α-dependent autophagy [31]. In BC, the activation of autophagic flux is an indicator of tumor regulation. AMP-activated protein kinase (AMPK) is a key energy sensor which can be modulated by cytokines such as leptin and adiponectin, and can promote autophagy and regulate cellular metabolism in maintaining energy homeostasis [32]. Chung et al. presented that adiponectin was a potent cytotoxic-autophagy inducer leading to BC inhibition and the mechanistic underpinnings involved in modulation of the STK11/LKB1 and AMPK-ULK1 axis [30]. Adiponectin markedly decreased growth and increased apoptosis in BC cells treated with various chemotherapeutic agents [30]. ULK1 is an autophagy-initiating kinase. Under nutrient sufficiency, a high mammalian target of rapamycin (mTOR) activity prevented ULK1 activation by phosphorylating ULK1 on Ser 757, thus destroying the interaction between ULK1 and AMPK [33]. Activated AMPK suppresses mTOR complex 1 (mTORC1) mediated activation of tuberous sclerosis 2 (TSC2) or inactivation of regulatory-associated protein of mTOR (Raptor) [34, 35]. The adipocyte-secreted adiponectin is a potent inducer of cytotoxic autophagy for BC inhibition, showing the therapeutic efficacy as a single agent or auxiliary anti-tumor substance.

Besides, adiponectin could also prevent the growth and invasion of BC cells by activating AMPK and inhibiting PI3K/AKT signaling pathway [13]. Adiponectin has a crucial role in obesity-associated BC. As previously documented, Mauro et al. found that adiponectin played an inhibitory role in ER-negative BC cell growth and progression in vitro and in vivo [36]. In contrast, low adiponectin levels was similar to those circulating adiponectin in obese patients, which was a growth factor for ER-positive BC cells that stimulated BC growth and progression [36]. Given that the downregulation of adiponectin in CAAs was similar to the mammary adipocytes in obese patients, low levels of CAA-secreted adiponectin might stimulate BC progression. It was of great interest that the adiponectin-to-leptin ratio derived from adipocytes in obese patients was different from that in normal people. Theriau et al. indicated that the adiponectin-to-leptin ratio was lower in obese patients, forming a growing environment that induced MCF-7 BC cells to enter the cell cycle [37]. This result was consistent with other studies that a high leptin-to-adiponectin ratio was associated with an increased risk of progression in postmenopausal BC and triple-negative BC (TNBC) [20, 38]. Hence, not only the increased adiponectin level but also the lower leptin-to-adiponectin ratio is associated with the decreased BC growth.

#### Il-6

Under pathological conditions like obesity and cancer, the level of IL-6 secreted by adipocytes is significantly increased. Among various cytokines secreted by adipocytes, the pleiotropic cytokine IL-6 is produced significantly, which is related to the development of stem cell phenotype, angiogenesis, cachexia, and resistance to therapy in BC [39]. IL-6 is identified as an independent adverse prognostic variable for overall survival and thereby is confirmed to be correlated with poor survival in hormone-refractory metastatic BC patients [40].

IL-6 expression is increased in CAAs and significantly boosts BC development. The study of Lee et al. showed that 3T3-L1 adipocytes indirectly co-cultivated with BC cells up-regulated the expression of inflammation-related genes IL-6 and Ptx3, which was consistent with the IL-6 overexpression observed in CAAs of human BC tissues [41]. Likewise, the secretion of adipocyte-derived IL-6 was increased when adipocytes were cocultured with MDA-MB-231 BC cells in vitro, while IL-6 blocking significantly decreased both the size and number of nodules in lung metastasis model of BC [24]. Fujisaki et al. isolated normal breast adipocytes and CAAs, and co-cultured them in collagen gels to mimic the in vivo environment, confirming the higher levels of IL-6 in the CAA-conditioned medium [12]. Their results also confirmed that IL-6 antibody neutralization abrogated the migration-enhancing effects of CAA-conditioned medium. Nickel et al. illustrated that after co-cultivation with adipocytes, migratory capacities were significantly increased in TNBC cells with the elevated secretion of IL-6, leading to an enhanced aggressive cell phenotype [42]. Adipocyte-derived IL-6 could also enhance the aggressive behavior of BC cells and induce EMT-phenotype [43]. Blocking the IL-6 signal in BC cells and adipocytes reduced proliferation, migration, and invasion capabilities of BC cells by altering the expression of EMT-regulating genes, destabilizing the focal adhesion and reducing cell motility [44].

#### CCL2 and CCL5

CCL2 is encoded by the CCL2 gene which is located on chromosome 17q12. Through binding to the G-protein-coupled receptors CCR2, CCL2 is a chemoattractant for recruiting CCR2-expressing immune cells to the inflammatory region [45]. CCL2 has been proposed as a target for metastatic BC because the high expression of CCL2 is correlated with a decrease in the survival of BC patients [46]. A neutralization experiment using antibodies against IL-6 or CCL2 showed the abolishment of migration-enhancing effects of the CAA-conditioned medium [12]. The result indicated that adipocytes could revert to an immature and proliferative phenotype in the presence of BC cells, and can promote cell migration via adipokines including IL-6 and CCL2 [12]. Hsieh et al. also found that adipocyte-derived CCL2 production was increased by co-culturing with 4T1 cells, and this phenomenon was broken by aspirin inhibition which might contribute to CCL2 chemo-preventive properties in BC [47]. Santander et al. reported that the expression of CCL2 was increased when E0771 BC cells were co-cultured with adipocytes and macrophages, thus recruiting more monocytes/macrophages in tumor progression [48]. In particular, there is the possibility that CCL2 plays a key role in the cross-talk between adipocytes and BC.

CCL5-CCR5 axis is related to the invasion and metastasis of BC. CCL5, initially termed as RANTES, is a potent chemokine that can attract leukocytes and a versatile inflammatory mediator expressed by BC cells [49]. Overexpression of CCL5 was correlated with ERK phosphorylation in tumor cells, and was statistically associated with poor disease-free survival and overall cancer survival in patients with early HER2-positive BC [50]. The abundance of CCL5 in peritumoral adipose tissues of TNBC patients is also correlated with metastasis and poor overall survival. Song et al. discovered that the elevated secretion of CCL5 in adipocytes co-cultured with TNBC cell lines heightened the EMT effect, thereby promoting the tumor growth, and lung and liver metastasis [51]. More importantly, another study showed that human adipocytes enhanced the invasiveness of MDA-MB-231 cells, and the antagonism of CCL5 with specific peptides and antibodies might reduce the invasiveness [52]. Therefore, antagonizing the expression of CCL2 and/or CCL5 in peritumoral adipocytes might be a novel therapeutic target for inhibiting BC growth and metastasis.

#### Other adipokines

In addition to the adipokines mentioned above, there are exceptional multiple adipokines involved in the interaction between CAAs and BC cells, including resistin, insulin-like growth factor 1 (IGF-1), hepatocyte growth factor (HGF), platelet-derived growth factor-BB (PDGF-BB), insulin-like growth factor binding protein 2 (IGFBP-2), IL-1β, IL-8, TNF-α, granulocyte colony-stimulating factor (G-CSF), vascular endothelial growth factor (VEGF) and autotaxin (ATX), lysophosphatidic acid (LPA). For example, resistin is an adipocyte-secreted factor elevated in BC patients. Lee et al. found that resistin promoted c-Src phosphorylation in BC, while either inhibition of c-Src and PKCα or knockdown of ezrin could block resistin-induced BC cell invasion [53]. In addition, Xiong et al. differentiated bone marrow-derived monocytic progenitors into adipocytes and co-injected these adipocytes with E0771 BC cells subcutaneously into C57BL/6 mice [54]. They verified that among the adipocyte-secreted adipokines, IGF-1, HGF and PDGF-BB contributed to the proliferation and migration of BC cells. It was reported that the IGFBP-2 was secreted by mature adipocytes around metastatic breast tumors compared with that in non-metastatic tumor tissue, resulting in the promotion of metastatic ability of MCF-7 cells [55].

The study of Kolb et al. found that IL-1β from primary adipocytes induced ANGPTL4 expression in a manner dependent on NF-κB- and MAP kinase-activation, leading to increased angiogenesis and BC progression [56]. IL-8 is a clinically relevant and promising therapeutic target for treating human BC. IL-8 released by mammary adipocytes induced a pro-tumoral activation of neutrophils, mechanistically accompanied by the overexpression of cell-adhesion molecules, which further increased the dissemination capacity of BC cells [57]. IL-8 plays a critical role in the activation of CAAs and serves as a main mediator for the paracrine pro-carcinogenic effects of mammary adipocytes. Besides, Al-Khalaf et al. successfully isolated CAAs from 10 invasive breast carcinomas, and found that all CAAs secreted higher levels of IL-8, which was crucial for mediating paracrine pro-carcinogenic effects of CAAs and enhancing the pro-angiogenic effects [58]. G-CSF has been found to be elevated and associated with various cancer types by binding to G-CSFR. Liu confirmed that CAA-derived G-CSF was critical for the invasive ability of BC, showing that targeting G-CSF/STAT3 signaling with G-CSF-neutralizing antibody could abrogate CAA-induced migration and invasion of BC cells [59]. In addition, co-culture of adipocytes and BC cells increased the secretion of VEGF and leptin, and thus enhanced the effects of estradiol and altered the risk of BC [60]. Co-cultured supernatants of adipocytes could also increase the proliferation, migration and sprouting of human umbilical vein endothelial cells (HUVECs), which might be ascribed to angiogenesis-associated VEGF [61]. Adipocytes, coupled with adipose-derived stem cells (ADSCs), are the main producers of ATX and LPA, which have attracted much attention in BC development [62]. Bidirectional interactions between BC cells and mammary adipocytes altered the local LPA axis and increased ATX expression in the mammary fat pad during ER-negative BC progression [63]. Therefore, targeting the ATX-LPA axis may represent an additive cancer therapy for invasive and metastatic tumors depending on the BC subtype.

### Metabolic reprogramming

Metabolic reprogramming, generally categorized as an emerging hallmark of cancer, is hijacked by BC to meet bioenergetic and biosynthetic demands, the redox balance maintenance, and cell proliferation [64]. The metabolic processes of BC and normal breast tissue are quite different in the aspect of glycolysis, tricarboxylic acid cycle, amino acids, nucleotides and/or lipid metabolism [65]. The nutrient transfer is an underlying mechanism in cancer-stromal cell crosstalk. Glutamine, the most abundant amino acid in the plasma, is the chief nutrient available to tumors, and is of great importance in tumor cell metabolism [66]. Recent studies have recognized that stromal cells could generate glutamine to promote cancer growth [67, 68]. It has been reported that adipocytes secreted glutamine and rescued pancreatic cancer cell proliferation in the absence of glutamine in vitro, suggesting that glutamine transfer is a potential mechanism in adipocyte-induced pancreatic cancer cell proliferation [68]. Glutaminolysis enables cancer cells to reduce NADP^+^ to NADPH, a reaction that is catalyzed by malic enzymes. Besides, NADPH is a required electron donor for reductive steps in lipid synthesis, nucleotide metabolism, and maintenance of reduced glutathione (GSH) [69]. CAAs play a functional role in tumor development through secreted adipocyte-derived factors and exosomes, and through metabolic symbiosis, in which malignant cells absorb lactic acid, fatty acids, and glutamine produced by adjacent adipocytes [70]. Therefore, it might be inferred that the metabolic reprogramming of glutaminolysis induced by CAAs could promote BC cells to regulate the redox state in order to favor proliferation.

Cancer cells prefer to produce adenosine triphosphate (ATP) by glycolysis, which is a less efficient pathway named as the “Warburg effect” compared to oxidative phosphorylation. The “reverse Warburg effect” is observed when cancer cells utilize the energy generated from stromal cells in the TME. In this theory, cancer cells can absorb free fatty acids (FFAs) and glycerol from interstitial adipocytes as energy sources [8, 71]. Adipocytes store triglycerides through adipogenesis, and produce diacylglycerols, monoacylglycerols and FFAs through lipolysis, thus regulating the energy balance of the entire organism [72]. Cancer-associated fibroblasts (CAFs), which are the main cellular members of the TME undergo metabolic reprogramming associated with the reverse Warburg effect [69]. Cancer cells create a “pseudo-hypoxic” microenvironment for CAFs. As HIF-1α promotes glycolysis and provides cancer cells with lactate and glutamate, increased reactive oxygen species (ROS) production in cancer cells induces the uptake of intermediate metabolites of the tricarboxylic acid (TCA) cycle in mitochondria indirectly. Cancer cell-derived ROS could down-regulate the expression of caveolin 1 (CAV1) in CAFs, and the loss of CAV1 also leads to increased ROS levels, thereby stabilizing HIF-1α [69]. The metabolic symbiosis between CAFs and epithelial cancer cells requires each other to express a different monocarboxylate transporter (MCT) isoform. MCT1 conduces to uptake the lactate produced by CAV1-deficient CAFs expressing MCT4, a marker of aerobic glycolysis and lactate efflux. Cancer cells then utilize the lactate to synthesize pyruvate which is an intermediate metabolite in the TCA cycle, while the acidic extracellular space rich in lactate, in turn, results in the formation of pseudo-hypoxic [73]. A previous study indicated that adipocytes adjacent to BC cells underwent phenotypic changes first marked by decreased adipocyte size and lipid content (CAAs), next by acquiring a fibroblast-like morphology (adipocyte-derived fibroblasts, ADFs), still with small lipid droplets. Then, ADFs exhibited increased migratory and invasive abilities and were able to join the center of the tumor, and these cells no longer expressed adipose markers nor exhibited lipid droplets, indistinguishable from other CAFs cell population [74]. Moreover, in addition to that CAAs possess a similar functionality with CAFs, both CAAs and CAFs participate in the complex and dynamic metabolic reprogramming to shape the TME in favorable conditions.

The continuous interaction between BC and adipocytes results in metabolic competition and symbiosis, leading to oncogenic-driven metabolic reprogramming of cancer cells and neighboring adipocytes. FFAs could be released over time from lipid droplets through an adipose triglyceride lipase (ATGL) dependent lipolytic pathway. ATGL was expressed in human BC and was up-regulated by the contact with adipocytes, thus boosting BC progression [75]. Through adipocyte-BC cell co-cultivation, Balaban verified that adipocyte-derived FFAs transferred into BC cells and drove fatty acid metabolism via increased CPT1A and electron transport chain complex protein levels, resulted in fueling BC proliferation and migration [76]. Lipid originated from tumor-surrounding adipocytes could be transferred into BC cells. Utilization of adipocyte-derived lipids and enhanced intracellular trafficking of fatty acids contributed to BC progression, by increased expression of the key lipase ATGL and intracellular fatty acid trafficking protein fatty acid-binding protein 5 (FABP5) [77]. Tumor lipid metabolism is regulated not only by genetic and epigenetic changes in the tumor cells but also by the availability of lipids provided by tumor-surrounding adipocytes. Zaoui et al. showed that mammary adipocytes potentiated the invasiveness of BC cells and the number of lipid droplets in adipocyte cytoplasm, which were mediated by metabolic reprogramming via CD36-mediated exogenous fatty acid uptake [78]. Wang et al. reported the function of the JAK/STAT3 pathway in regulating lipid metabolism, once inhibiting JAK/STAT3 would lead to the block of diverse lipid metabolic gene expression, including CPT1B that encoded the critical enzyme for FAO [25]. Mammary adipocyte-derived leptin could also down-regulate CD8^+^ T cell effector functions through activating STAT3-FAO and inhibiting glycolysis in promoting obesity-associated breast tumorigenesis [27].

In addition to BC cell reprogramming induced by CAAs, adipocytes also undergo reprogramming to become CAAs under the stimulation of BC cells in reverse. Cytokines or exosomal compositions (proteins, miRNAs, lncRNAs and circRNAs) are probably exploited by BC cells as a sort of “signal” to convert the cells in TME such as adipocytes and CAFs into a pro-tumor niche. For example, Wu et al. confirmed that exosomes containing miRNA-144 and miRNA-126 were highly secreted from BC cells co-cultured with adipocytes, leading to promoted metastasis by inducing beige/brown differentiation and reprogramming the metabolism in surrounding adipocytes in vivo [79]. They indicated a new mechanism that tumor-adipocyte interaction reprogramed systemic energy metabolism to facilitate tumor progression [79]. Lee et al. also confirmed the importance of miRNA-regulatory mechanisms of the transition into inflammatory CAAs in BC microenvironment [41]. In this study, mmu-miR-5112 showed the highest expression in the preadipocyte state and the expression was suppressed during the process of adipocyte differentiation. Mmu-miR-5112 mediated the regulation of IL-6 in CAAs, which presented de-differentiated and inflammatory natures in BC TME [41]. This miRNA-based regulatory mechanism involved acquiring the inflammatory phenotypes of CAAs. In addition, using specific shRNA, anti-IL-8 antibody, or reparixin, Al-Khalaf et al. found that the IL-8 signaling inhibition suppressed the active features of CAAs features, including non-cell-autonomous tumor-promoting activities both on breast luminal cells and in orthotopic tumor xenografts in mice [58]. It provided a clear indication that IL-8 was a critical factor in the activation of breast CAAs. Wei et al. reported that plasminogen activator inhibitor type 1 (PAI-1), which had been implicated as a pro-tumorigenic agent in cancers, particularly in cancer metastasis, was required to activate the expression of the intracellular enzyme PLOD2 in CAAs [80]. Furthermore, PLOD2-producing CAAs could remodel collagen alignment during crosstalk with BC cells and further promoted BC metastasis [80]. Therefore, these studies have indicated that there is a complex metabolic network favoring malignant progression that is established between BC cells and adipocytes at the tumor invasive front. The interaction entails a vicious circle, wherein BC cells reprogram adipocytes, which in turn promote tumor progression. Specific factors, such as inflammatory factors, lncRNAs, exosomal compositions and adrenomedullin, have been demonstrated to be involved in this process.

### Extracellular matrix remodeling

Adipose tissue is surrounded by a basement membrane, which is mainly composed of collagen IV, laminin and fibronectin. Components released by adipocytes possess the capability of remodeling ECM and shaping the BC course [8]. ECM adaptation is essential for the progression and invasion of BC, which could be possibly altered by adipocytes. Interestingly, in an accessible 3-dimensional (3D) co-culture system, Pallegar et al. demonstrated that adipocytes promoted a mesenchymal-to-epithelial transition (MET)-like change in mesenchymal TNBC cells, emphasizing the reciprocal interaction between high adiposity and BC metastasis with adipocyte abundance [81].

Adipocytes in BC can affect the synthesis of matrix proteins and the activation of matrix-related enzymes. Adipocytes can secrete multiple components orchestrating for ECM mechanical support. An immunostaining study of the clinical samples represented that the metastatic human BC tumors had higher levels of matrix metalloproteinase (MMP)-2, compared with non-metastatic tumor tissues; whereas adipocytes around metastatic BC tumors had higher levels of IGFBP-2 than that of non-metastatic sites [55]. Furthermore, co-culture media of mature adipocytes and MCF-7 BC cells showed remarkably up-regulated MMP-2 and enhanced invasion ability of MCF-7 cells [55]. Juárez-Cruz et al. proved that adipocyte-derived leptin promoted the secretion of ECM remodelers, MMP-2 and MMP-9 in a FAK and Src-dependent manner, thereby degrading collagen IV and promoting the rupture of basement membranes [23]. The results suggested that leptin promoted BC into a more aggressive invasive phenotype. Furthermore, another study showed that MMP-2 and MMP-9 suppression could result in the inhibition of BC invasion via emodin by down-regulating the level of CCL5 from adipocytes. It was meaningful to antagonize the secretion of CCL5 from adipocytes for inhibiting BC metastasis. Similarly, adipocytes cultivated with cancer cells exhibited an altered phenotype including MMP-11 overexpression, and played a key role in the acquired pro-invasive effect of tumor cells [14, 82].

Adipocytes also played a vital role in tumor growth at early stages through secretion and processing of collagen VI [83]. Collagen VI, a soluble ECM protein abundantly expressed in adipocytes and enriched in BC lesions, is a key protein for BC ECM. By secreting and processing collagen VI, adipocytes were essential in defining the ECM environment for normal and tumor-derived ductal epithelial cells and contributed significantly to tumor growth [83]. Park et al. investigated that endotrophin, which was derived from collagen VI cleaved by MMP-11, showed a strong stimulating effect on the growth, angiogenesis, and tissue fibrosis of BC [84]. Besides, endotrophin acted as a stimulator to promote the EMT through enhanced TGF-β signaling, contributing to aggressive and high metastatic BC growth [84].

A collagen-dense ECM can potently interact with hormonal signals to drive invasion and metastasis of ER-positive BC [85]. PLOD2 participated in collagen synthesis. Accordingly, the research of He et al. clarified that adipocyte-derived IL-6 and leptin could promote PLOD2 expression by activating the JAK/STAT3, thus facilitating the hardness and metastasis of BC [24]. By constructing a 3D collagen invasion model, Wei et al. showed that CAAs remodeled collagen alignment concerning of PAI-1 or PLOD2 up-regulation during crosstalk with BC cells in vitro and in vivo, further boosting BC metastasis [80]. It is worth noting that the increased matrix stiffness, which can be sensed by many cell types, is a feature of most solid tumors [86]. Yes-associated protein (YAP) function is essential to establish and maintain the tumor-promoting function of CAFs, including stromal sclerosis, invasion and angiogenesis. The high level of YAP in the matrix is maintained by the positive feedback between CAF-driven matrix hardening and mechanical conduction, leading to YAP activation [87]. It has been reported that the Hippo pathway can be activated by stromal stiffness in solid tumor tissues, and central to this signaling is a kinase cascade leading from the tumor suppressor Hippo to the oncogenic Yki (YAP/TAZ in mammals), which is a transcriptional coactivator of target genes involved in cell proliferation and survival [88]. In addition, YAP is activated in CAFs in response to mechanical stress, perturbation of the actin cytoskeleton and ECM stiffness [86, 87]. Besides, the increased expression of YAP in preadipocytes is correlated with the repression of adipogenic differentiation processes [89]. Wang et al. conducted a transcriptomic analysis to find that mesenchymal stromal/stem cell (MSC)-differentiated adipocyte exosomes activated the Hippo signaling pathway involved in two key downstream Hippo proteins YAP and TAZ in MCF-7 cells, while the Hippo pathway blockade could lead to reduced tumor growth [90]. This result mechanistically emphasized that the Hippo signaling pathway was partially responsible for the tumor-promoting effects of MSC-differentiated adipocyte exosomes [90]. Therefore, YAP-dependent matrix stiffening driven by CAFs or YAP activation induced by adipocytes, might lead to pro-tumorigenic YAP activation in BC cells in close proximity to the stiff matrix.

Therefore, these studies demonstrate that adipocyte-derived factors exert roles in ECM architecture remodeling for facilitating the BC cell migration and motility. Abrogating CAA-dependent ECM re-organization of BC will decrease the directional migration of cancer cells within the matrices, thus leading to favorable outcomes.

### microRNAs

miRNAs are a unique class of endogenous small, short single-stranded, and non-coding RNA molecules. The most important function of miRNAs is to negatively regulate numerous mRNAs by silencing their target transcripts. Accumulating evidence indicates that the expression of miRNAs during BC progression is aberrantly altered while several miRNAs are recognized to be crucial regulators of adipose microenvironment [91, 92]. Rajarajan et al. found that in vitro co-culture of BC cells with mature adipocytes increased proliferation, migration, and invasive phenotype of BC cells, and 98 miRNAs were differentially expressed in BC cells by using small RNA-sequencing analysis [93]. Among them, miR-3184-5p and miR-181c-3p were found to be the most up-regulated and down-regulated miRNAs, and direct targets were FOXP4 and PPARα, respectively. It was hypothesized by Lee et al. that mmu-miR-5112 treated-adipocytes showed up-regulation of IL-6 by targeting Cpeb1, proposing a miRNA-based regulatory mechanism underlying the process of acquiring inflammatory phenotypes in CAAs [41]. Wu et al. revealed that tumor cells co-cultivated with mature adipocytes exhibited an aggressive phenotype through inducing EMT, while BC cell-secreted miR-155 promoted beige/brown differentiation and remodeled metabolism in resident adipocytes [94]. Interactions between adipocytes and BC cells stimulated cytokine production and drove src/sox2/mir-302b-mediated malignant progression [95]. Besides, miR-141 and miR-146b-5p were two important tumor suppressor miRNAs in BC, and mediated the p16-negative regulation of leptin in adipocytes [96]. In summary, miRNAs are key factors and mediators in intercellular communication. In the progress and metastasis of BC, the miRNA-based regulatory mechanism is closely related to the adipocyte-secreted factors.

### Immune cells

The peritumoral adipocytes induce an immune remodeling in favor of cancer aggressiveness and progression, mainly including cells of monocytes, macrophages, neutrophils and T cells [97, 98]. Crown-like structures (CLSs), composed of macrophages encapsulating dead or dying adipocytes, are a histologic hallmark of the pro-inflammatory process. In this process, adipose tissue contributes to the increased risk and worse prognosis of BC in obese, postmenopausal patients as well as some normal-weight women [99]. CD68^+^ and/or CD163^+^ TAM infiltration, coupled with CLSs, is present in adipose tissue nearby the BC lesion, and is associated with various clinicopathologic parameters of BC [17]. Besides, the adipocyte-derived chemokines, such as CCL2, recruited macrophages to the TME and further triggered BC development [48, 84, 100, 101]. In addition, adipokines are able to induce angiogenesis in driving tumor progression through macrophages. Several adipokines including leptin, IL-6, and TNF-α are capable of increasing VEGFA expression in THP-1 macrophages co-cultured with adipocytes. Compared with THP-1 cells cultured alone, the medium of THP-1 cells previously exposed to human adipocytes stimulated endothelial tube formation more significantly [102]. Therefore, revealing the underlying mechanisms of adipocyte-driven immune cells and immune cascades is necessary to understand the role of adipokines in TME.

## Adipocytes in breast cancer therapy

In addition to promoting BC aggressiveness, adipose secretome and cellular interactions are implicated in the resistance to multiple therapies, including chemotherapy, hormonal therapy, radiotherapy and immunotherapy. The known drug-resistant mechanisms mainly include activation of drug transporter proteins, evasion of therapy-induced apoptosis, enhanced DNA repair mechanisms and alterations of drug metabolism [103].

Indeed, adipose microenvironment has been described to influence the anti-cancer efficiency of chemo- and hormonal therapies (doxorubicin, tamoxifen and fulvestrant). Tumor-surrounding adipocyte-stimulated chemoresistance has been corroborated by lots of studies. Major vault protein (MVP) is a transport-associated protein with up-regulated expression in BC mediated by adipocytes. Cocultivation with human mammary adipocytes induced chemoresistance in BC cells, manifested as a result of altered subcellular distribution and excretion from the BC cell membrane treated with doxorubicin. The result was a piece of evidence that adipocytes induced an MVP-related multidrug-resistant phenotype, which could contribute to obesity-related chemoresistance [104]. Sequester and efficient metabolism of a pharmaceutical agent by adipocytes could be clinically important for adipocyte-riched cancer microenvironments such as omentum, breast, and marrow. Sheng et al. pointed out that adipocytes metabolized daunorubicin to a less toxic metabolite, and allowed nearby acute lymphoblastic leukemia cells to evade daunorubicin-induced cytotoxicity [105]. It is known that cancer stem cells (CSCs) are metabolically distinct from cancer cells and are critical for chemoresistance. Wang et al. found that the mammary adipocyte-derived leptin promoted cancer cell stemness, and thus enhanced the chemoresistance by activating STAT3-CPT1B-FAO in breast CSCs [25]. This study identified a critical strategy that blocking FAO and/or leptin re-sensitized the BC cells to chemotherapy. Besides, adipokines, such as leptin, were able to weaken the anti-tumor effect of hormonal tamoxifen therapy [106]. For instance, Bougaret et al. investigated that leptin, IL-6 and TNF-α in obese patients decreased the anti-proliferative efficacy of 4-hydroxytamoxifen, which was a major active metabolite of tamoxifen [107]. In overall and tamoxifen-treated BC patients, leptin expression correlated with poor prognosis [108].

It is also of interest that the interactions between adipocytes and BC cells participate in the resistance of radiotherapy and immunotherapy. In a vitro assay, Bochet et al. showed that BC cells exhibited enhanced IL-6 expression, Chk1 phosphorylation, and a radioresistant phenotype in the presence of adipocytes, revealing a new role of tumor-surrounding adipocytes in fostering a radioresistant phenotype in BC [109]. Tang et al. reported that the mammary adipocyte-derived ATX secretion was promoted by BC-produced inflammatory cytokines, leading to enhanced resisting effects in radiotherapy or chemotherapy [110, 111]. Thus, inhibiting adipocyte-derived ATX provided a promising adjuvant to improve BC outcomes of radiotherapy and chemotherapy.

Acquired resistance to trastuzumab is a clinical problem in the treatment of HER2-over-expressing metastatic BC. Adipocyte-secreted factors such as growth differentiation factor 15 (GDF15), resulted in reduced response to trastuzumab by stimulating PI3K signaling [112]. Duong et al. also proved that adipocytes could promote resistance of HER2-expressing BC cells to trastuzumab-mediated antibody-dependent cellular cytotoxicity, via reducing interferon (IFN)-γ secretion by NK cells [113]. Besides, Wu et al. showed that adipocyte-secreted PD-L1 could prevent the anti-PD-L1 antibody from activating important antitumor functions of CD8^+^ T cells [114]. Hence, pharmacologic inhibition of adipogenesis selectively reduced PD-L1 expression in murine adipose tissue and enhanced the antitumor efficacy of anti-PD-L1 or anti-PD-1 antibodies in syngeneic mammary tumor models.

Collectively, it is important to underline the utility of adipokines as predictors for drug resistance and the role of adipose tissue in the resistance to BC therapy (Table 2). Approaches targeting the adipocyte and cancer cell crosstalk may help sensitize cancer cells to BC therapy, deserving further examined.

**Table 2:** The impact of adipocytes on breast cancer therapy

Therapeutic type	Therapeutic drug	Cancer cell type	Mechanism of therapeutic resistance	Ref.
Chemotherapy	Doxorubicin	T47D, MDA-MB453, BT-474, MDA-MB436, MDA-MB231, M-Wnt and E0771	Adipocytes induced an MVP-related DOX-resistant phenotype in BC cells	[104]
	Paclitaxel	MDA-MB-231	Mammary-adipocyte-derived leptin upregulated CPT1B expression and FAO activity in BCSCs, thus promoting cancer cell stemness and chemoresistance	[25]
Hormonal therapy	Tamoxifen	MCF-7, MDA-MB-231, T47D and MDA-MB-435	Mature adipocytes and their secretions were able to increase mammary cancer cell proliferation and to suppress the antiproliferative effect of tamoxifen	[106–108]
Radiotherapy	–	SUM159PT	Tumor-surrounding adipocytes fostered a radioresistant phenotype in breast tumors through the increasing expression of IL-6 in tumor cells	[109]
	–	Hs578T and 4T1	Inflammatory cytokines produced by tumors increased ATX secretion of mammary adipocytes, and enhanced the ATX-LPA inflammatory cycle, thus resisting radiotherapy	[110, 111]
Immunotherapy	Trastuzumab	BT-474, SK-BR-3, MDA-MB-453, and MDA-MB-361	Adipocytes inhibited trastuzumab-mediated ADCC in HER2-expressing BC cells via the secretion of soluble factors	[113]
	Anti-PD1/PD-L1 antibody	E0771	Adipocyte PD-L1 prevented anti-PD-L1 antibody from activating important antitumor functions of CD8^+^ T cells	[114]

Abbreviations: *DOX* doxorubicin; *MVP* major vault protein; *BC* breast cancer; *BCSCs* breast cancer stem cells; *CPT1B* carnitine palmitoyltransferase 1B; *FAO* fatty acid β-oxidation; *ADCC* antibody-dependent cellular cytotoxicity; *HER2* human epidermal growth factor receptor 2

## Adipocytes in autologous fat grafting of breast reconstruction

Autologous fat grafting is becoming an increasingly attractive procedure for breast reconstruction in BC patients who have undergone a mastectomy. The fat donor, harvested by liposuction, is transplanted into the breast to obtain a better breast morphology. Cell-assisted lipotransfer (CAL) is a process in which fat grafting is supplemented with autologous ADSCs, and can reduce the fat absorption rate and improve the survival rate of fat grafting [115]. Regarding the contribution of CAAs to the progress of BC, oncologic safety of breast lipofilling after a mastectomy is inevitably a major clinical issue. Even though the breast tumor of BC patients receiving fat grafting is removed, it still exists the possible presence of incipient in situ lesions or residual dormant tumor cells [13].

Previous studies have confirmed the role of adipocytes and ADSCs in promoting BC in a cell model and in vivo. In clinical studies, the risk of local recurrence in BC patients receiving autologous fat grafting after mastectomy remains in a twilight zone. Interestingly, Gebremeskel et al. reported that ADSCs fat grafting alone, but not conventional fat graft or cell-assisted lipotransfer, could promote BC cell proliferation and invasiveness in vitro and in mouse model [116]. The possible potential reasons were that the fat might act as a barrier to prevent ADSC-produced soluble factors from reaching cancer cells, and co-injected fat might exert a paracrine influence on ADSCs, causing them to preferentially undergo adipogenesis as opposed to angiogenesis. Cohen et al. indicated that tumor recurrence rate in the autologous fat grafting group was 2.5%, which was no significant difference with the control group, while the mean time to recurrence in the fat grafting group was significantly longer than that in the control group [117]. This provided valuable evidence-based support for oncologic safety of fat grafting. Similarly, in a case-controlled study involved in 205 patients with fat grafting reconstruction after BC surgery, the results showed that BC recurrence was not increased with lipofilling reconstruction [118].

**Fig. 2:**
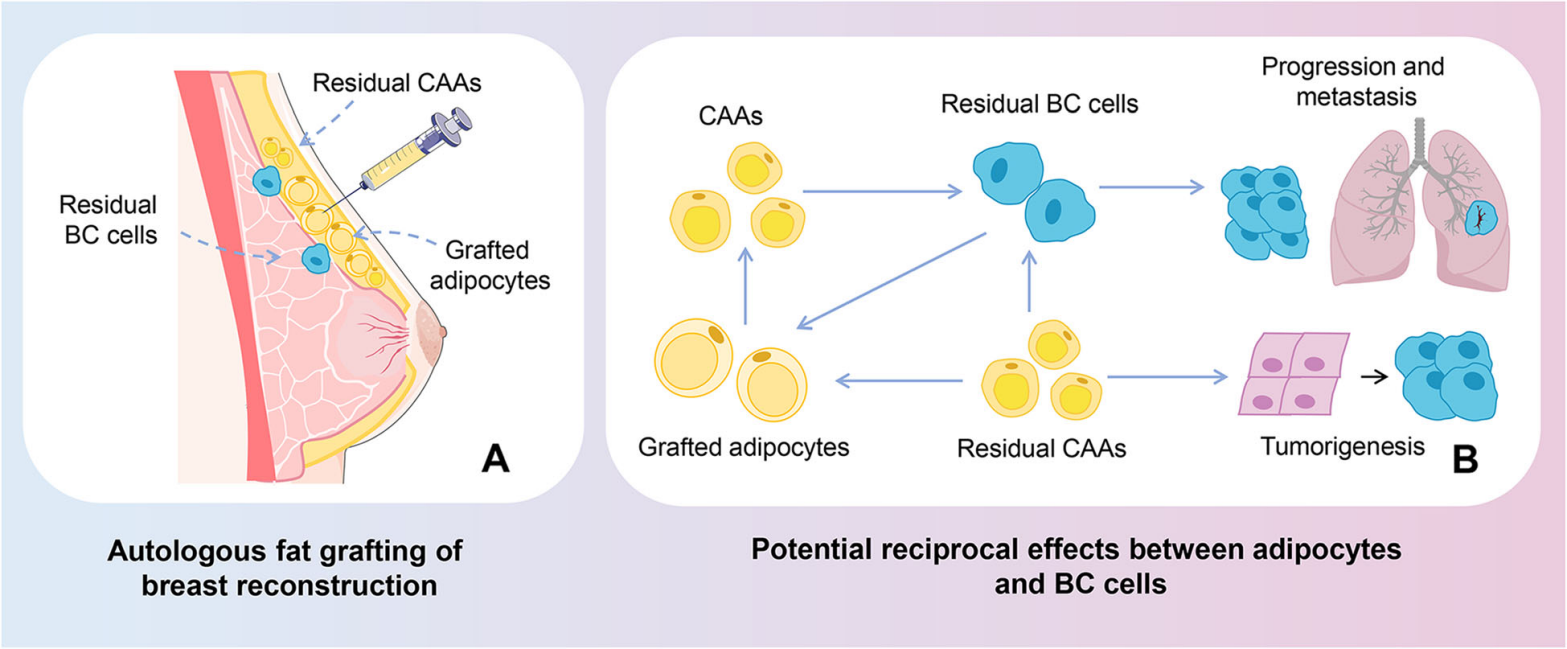
Oncologic safety in autologous fat grafting of breast reconstruction. **a** Autologous fat grafting of breast reconstruction. Autologous fat grafting is a method of breast reconstruction after mastectomy. Although the breast tumor of patients undergoing fat grafting has been removed, there still exists the possibility of incipient in situ lesions or residual dormant tumor cells or residual CAAs. **b** Potential reciprocal effects between adipocytes and BC cells. The residual BC cells may interact with the grafted adipocytes and shift them into CAAs, resulting in progression and metastasis of BC. Besides, residual CAAs may also impact on the residual BC cells in promoting BC progress, or may play a role in tumorigenesis of BC. Residual CAAs might affect the grafted adipocytes and further enhance the pro-carcinogenic effects of adipocytes. Abbreviations: BC, breast cancer; CAAs, cancer-associated adipocytes

Despite breast reconstruction using fat grafting is a standardized and widely popularized technique, evidence of oncological safety still deserves consideration. It is necessary to conduct clinical trials on a large scale and with long-term follow-up in order to ensure the oncologic safety of fat grafting after mastectomy. Besides, BC patients who needed autologous fat grafting should be strictly screened, and the potential recurrence should be closely observed after surgery. Patients with a high risk of BC recurrence should avoid or postpone autologous fat grafting and avoid injecting high purity ADSCs (Fig. 2).

## Conclusions

Collectively, adipocytes are excellent candidates to modify tumor behavior through heterotypic signaling processes by the secretion of adipokines like hormones, growth factors, cytokines and other molecules. From the established evidence in vitro, in vivo and in clinical, it can be introduced as follows: (i) the characterization of factors derived from adipocytes is changed during tumor progression and is confirmed in human BC samples. (ii) BC cells remarkably impact on surrounding adipocytes; (iii) peritumoral adipocytes exhibit a modified phenotype and specific biological features sufficient to be named CAAs; and (iv) CAAs alter the BC cell characteristics/phenotype leading to more aggressive behavior. Therefore, it can propose that adipocytes are the participators in a highly complex, inflammatory cycle orchestrated by BC cells to promote tumor progression which might be amplified in CAAs vicious function. Hereby, CAAs may become an obstacle to BC therapy. CAA-caused resistance in BC therapy, such as chemotherapy, radiotherapy, hormonal therapy and immunotherapy, might lead to persistent tumor residue and increased risk of recurrence.

Nevertheless, there are still some challenges in the identification for CAAs. At present, some CAA-related genes and protein markers have been identified but there is not a uniform certification standard of CAAs. Thus, CAA-related characteristics need to be further established. Significant effort based on CAAs should be invested to identify biomarkers for the diagnosis, prediction of therapy response, and prognosis of BC. The complicated mechanisms are involved in CAAs of BC, including the secretion of adipokines, metabolic reprogramming, ECM remodeling, miRNAs and immune cells regulating. However, these potential mechanisms of CAAs remain largely elusive. For example, Ryu et al. concluded a contrary result in the prevailing concept of the cancer-promoting role of CAAs, presuming the interaction between BC and CAAs is rather complex [119]. Besides, some adipokines, like IL-6, are of paradoxical effect on promoting or inhibiting in different BC courses. Deciphering this complex network is critical to improving the treatment outcomes and the ultimate survival rate of patients.

Another important issue is the adipokine source in TME. Multiple cell types and soluble active factors comprise the intricate TME of BC. It poses an issue that specific adipokines such as polyfunctional IL-6 could be released by different cell types, primary adipocytes, immune cells, or tumor cells. It would be an interesting angle that the specific factor is secreted by which cell type. Besides, the IL-6R is also expressed in these cell types, proposing the inclination of an autocrine or paracrine function. Although clinical specimens verified the correlation between certain CAA-factors and BC, the tumor-promoting effect of CAAs is often determined by cell experiments in vitro, but not animals.

It will be meaningful to take consideration of CAA functions to the BC therapy tackled by researchers. The intervention between CAAs and BC cells might be a potential application in auxiliary therapy against BC development. It is noteworthy that the growth and metastasis characteristics of BC cells are closely related to BC subtypes. The existing studies on CAAs in BC mainly focus on BC subtypes as follows: ER-positive BC, ER-negative BC, TNBC, non-TNBC, Luminal A and other unspecific BC subtypes (as described in Table 3). In all these BC subtypes, CAAs exhibit the ability to promote BC progress via their paracrine functions. CAAs are able to stimulate the migration and invasive capacities of ER-positive and negative BC, and the promoting function seems to be more profound in ER-negative BC compared with ER-positive BC. In terms of the proliferation-promoting effect of CAAs, it is controversial to indicate whether ER-negative BC or ER-positive BC is more obvious. Especially, TNBC accounts for nearly 15% of all invasive BC, and possesses the highest rate of metastatic occurrence and the poorest overall survival in all BC subtypes. CAAs could change TNBC cells into more aggressive phenotypes with the enhanced ability of migration and invasion. Further studies are still needed to deeply uncover the underlying mechanisms of CAAs for a better comparison of different BC subtypes. The BC subtype together with different tumor processes would exert a certain impact on CAA-targeted therapy. Implementing other strategies designed to target the adipokine receptors might be beneficial to modulate the sensitivities of CAA-derived adipokines in reducing the pro-oncogenic function of CAAs, especially for metastasis. In particular, residual adipose tissue around BC tumor does make sense on the influence of recurrence after mastectomy, further affirming the clinical value of the precise resection boundary of adipose tissue. In addition, fat grafting may complicate breast imaging and BC surveillance because of fat necrosis and calcification in breast tissue. It is even more remarkable for plastic surgeons that whether the CAA-containing residual adipose tissue could lead to BC recurrence, and whether the residual CAAs could affect the grafted adipocytes in the process above.

**Table 3:** The impact of CAAs on breast cancer subtypes

Breast cancer subtype	Model	Impact of CAAs on breast cancer subtypes	Ref.
ER-positive	Co-culture model (MCF-7)	A direct co-culture between murine 3T3-L1-derived adipocytes and BC cells of various molecular subtypes induced an EMT phenotype and enhanced their proliferation, migration, and invasion capabilities.	[11]
ER-negative	Co-culture model (MDA- MB-231, MDA-MB-468)		
ER-positive	Co-culture model (MCF-7)	CAA-conditioned medium increased the cell migration of both MCF-7 and MDA-MB-231 cells via IL-6 and CCL2.	[12]
ER-negative	Co-culture model (MDA-MB-231)		
ER-positive	Co-culture model (ZR 75.1)	Mature adipocytes were able to stimulate the invasive capacities of murine and human BC cell lines that are either positive (ZR 75.1) or negative (SUM159PT, 67NR, 4 T1) for the ER. Coculture with adipocytes had no effect on ZR 75.1, 67NR or 4T1 proliferation in contrast to SUM159PT cells.	[14]
ER-negative	Co-culture model (SUM 159PT, 67NR, 4T1)		
Luminal A	Co-culture model (MCF-7); human invasive ductal carcinoma sample	A total of 1126 and 1218 proteins were identified in MCF-7 and MDA-MB-231 cells, respectively. Among these, 85 (MCF-7) and 63 (MDA-MB- 231) had an average fold change > 1.5 following co-culture.	[16]
TNBC	Co-culture model (MDA-MB-231); human invasive ductal carcinoma sample		
TNBC	Co-culture model (MDA-MB-231, MDA-MB-468); clinical samples in the dataset	Adipocytes could facilitate the pro-metastasis role in TNBC and non-TNBC via PLOD2-dependent way. PLOD2 expression was much higher in TNBC patients, compared to non-TNBC patients.	[24]
non-TNBC	Co-culture model (SK-BR-3); clinical samples in the dataset		
ER-positive	Co-culture model (MCF-7, ZR-75-1)	The transition of adipocytes into more inflammatory CAAs resulted in proliferation-promoting effect in ER-positive BC cells such as MCF7 and ZR-75-1 but not in ER-negative cells; aromatase levels were upregulated in CAAs that might favor the growth of ER-positive BC cells.	[41]
ER-negative	Co-culture model (MDA-MB-231, Hs578T)		
Not-given	Human BC samples	The expression of IL-6 was up-regulated in CAAs in human BC tissues.	
ER-positive	Co-culture model (MCF-7, T47D)	In ER-positive cell lines, top upregulated genes showed significant enrichment for hormone receptor target genes. In triple-negative MDA-MB-231 cells, co-culture with adipocytes led to the induction of pro-inflammatory genes, mainly involving genes of the Nf-κB signaling pathway, and increased secretion of the pro-inflammatory interleukins IL-6 and IL-8.	[42]
TNBC	Co-culture model (MDA-MB-231)		
Luminal A	Co-culture model (MCF-7)	Human adipocytes could enhance proliferation, migration and invasion abilities of MDA-MB-468 and MCF-7 cells after co-culture, and these effects were more profound in MDA-MB-468 cells compared with MCF-7, which are non-invasive cells.	[43]
TNBC	Co-culture mode (MDA-MB-468)		
TNBC	Co-culture model (MDA-MB-231, MDA-MB-453)	Elevated secretion of CCL5 in adipocytes co-cultured with the TNBC cell lines heightened the EMT effect, thereby promoting tumor growth and lung and liver metastasis.	[51]
TNBC	Co-culture model (E0771); in vivo mouse orthotopic tumor model	Conditioned media from adipocytes supported E0771 cell proliferation and enhanced cell migration in vitro; adipocytes not only accelerated breast tumor growth, but also enhanced vascularization in vivo.	[54]
ER-positive	Co-culture model (MCF-7)	The co-culture media of human MCF-7 BC cells and human mature adipocytes increased the motility of MCF-7 cells through IGFBP-2.	[55]
Not-given	10 metastatic pathologic samples and 10 non-metastatic pathologic samples	Metastatic human breast tumors had higher levels of MMP-2 than did non-metastatic tumor tissue, whereas adipocytes around metastatic breast tumors had higher levels of IGFBP-2 than did adipocytes surrounding non-metastatic breast tumors.	
ER-positive	In vivo zebrafish model (MCF-7, T47D)	Breast adipocyte increased the dissemination of ER-positive BC cells in the zebrafish model of metastasis, while dissemination of the more aggressive and metastatic BC cells such as ER-negative was unaffected.	[57]
ER-negative	In vivo zebrafish model (MDA-MB-231)		
Not-given	Human invasive ductal carcinoma sample	CAAs isolated from 10 invasive breast carcinomas were pro-inflammatory and exhibited active phenotypes, including higher proliferative, invasive and migratory capacities.	[58]
TNBC	Co-culture model (MDA-MB-231, BT549)	CAAs could enhance migration and invasion of TNBC cells, while the effect of CAAs on ER-positive BC cells were limited.	[59]
ER-positive	Co-culture model (MCF-7, T47D)		
Not-given	Human BC tissue sample	Elevated G-CSF expression in adipocytes was well correlated with activated Stat3 signal in cancer cells.	
ER-negative	Co-culture model (M28N2, M27H4, M6)	Mammary adipose tissue-derived lysophospholipids promoted ER-negative mammary epithelial cell proliferation.	[63]
Luminal A	Co-culture model (MCF-7)	Proliferation, migration and invasion were increased in BC cells, which was most prominent for the highly invasive SUM159 cells and, to a lesser extent, for the less invasive MCF7 cells.	[78]
TNBC	Co-culture model (SUM159)		
Not-given	Human invasive breast carcinoma sample	Collagen reorganization at the tumor-adipose periphery, as well as the positive relevance between PAI-1 and PLOD2 were found in invasive breast carcinoma, revealing a new stromal collagen network that favors tumor invasion and metastasis establish between BC cells and surrounding adipocytes at the tumor invasive front.	[80]
TNBC	Co-culture model (MDA-MB-231, Hs578t)	Adipocytes and adipocyte-derived conditioned media, but not pre-adipocytes, caused the mesenchymal MDA-MB-231 and Hs578t cells to form significantly more epithelial-like structures when compared to the typical stellate colonies formed in control 3D cultures. MCF7 cells had a less dramatic shift as they normally have a more epithelial-like structure in 3D culture.	[81]
Luminal A	Co-culture model (MCF7)		
ER-positive	Co-culture model (T47D)	Adipocytes caused DOX resistance in all the cell lines studied, independently of the BC subtypes.	[104]
HER2-positive	Co-culture model (MDA-MB453, BT-474)		
TNBC	Co-culture model (MDA-MB436, MDA-MB231, M-Wnt, E0771)		

Abbreviations: *BC *breast cancer; *ER* estrogen receptor; *TNBC* triple-negative BC; *IL-6* interleukin 6; *IL-8* interleukin 8; *CCL2* chemokine ligand 2; *CCL5* chemokine ligand 5; *CAAs* cancer-associated adipocytes; *EMT* epithelial-mesenchymal transition; *IGFBP-2* insulin-like growth factor binding protein 2; *G-CSF* granulocyte colony-stimulating factor; *DOX* doxorubicin; *PLOD2* lysyl hydroxylase 2; *PAI-1* plasminogen activator inhibitor type 1

**Fig. 3:**
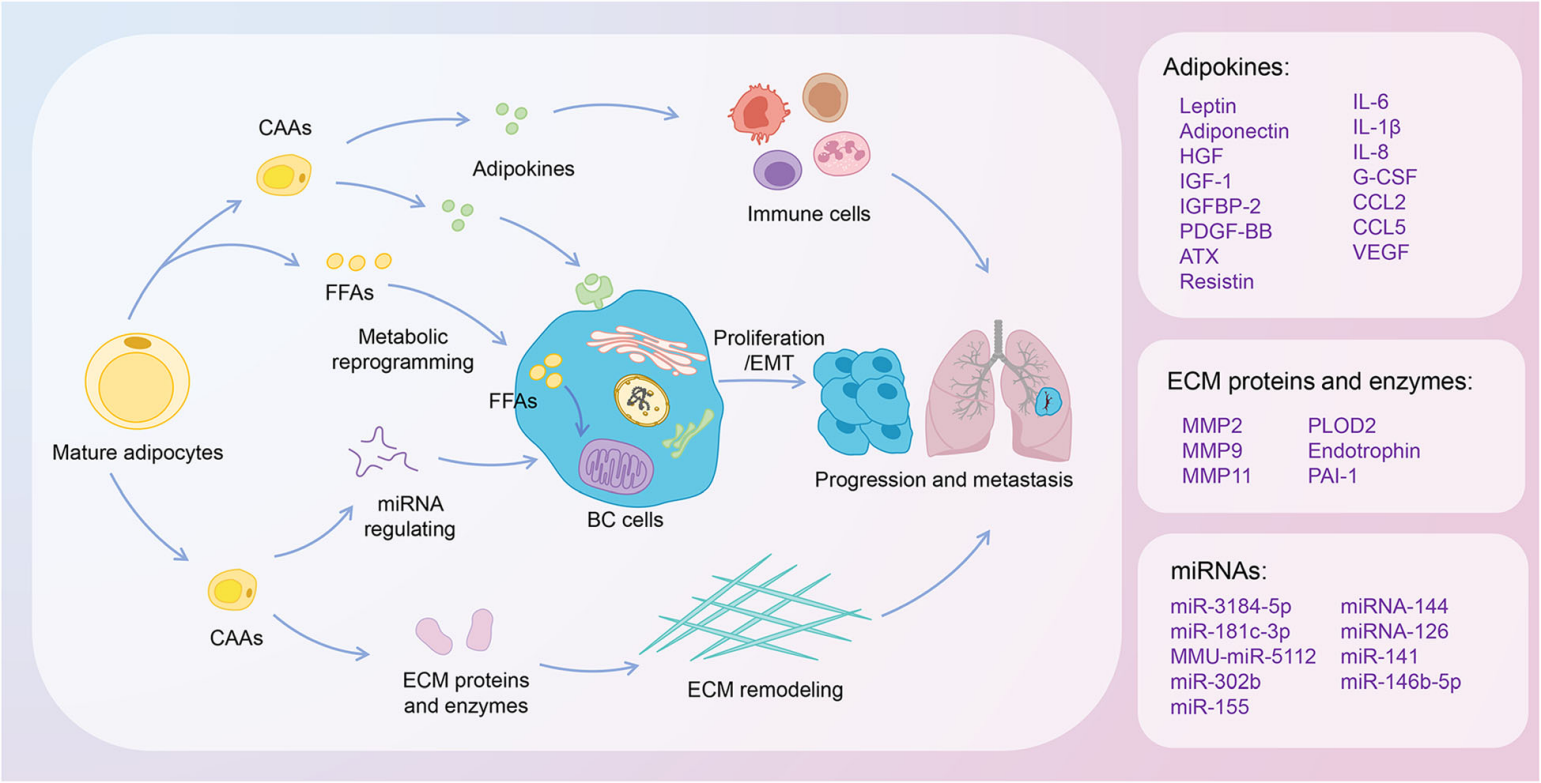
Mechanisms of adipocytes in regulating the progression and metastasis of breast cancer. In invasive front of BC cells, adipocytes undergo lipolysis and change into an active phenotype, referred to as CAAs. CAAs are involved in the process of tumor progression and metastasis of BC, including secretion of multiple adipokines; ECM remodeling by expressing and activating of several ECM proteins and enzymes; metabolic reprogramming by transferring FFAs, released in the lipolysis process of adipocytes, into the BC cell and increasing FAO; miRNA regulating; immune cells. Abbreviations: BC, Breast cancer; CAAs, cancer-associated adipocytes; ECM, extracellular matrix; FFAs, free fatty acids; FAO, fatty acid β-oxidation; EMT, epithelial-mesenchymal transition

Thus, we offer the key insights into the potential functions and mechanisms of CAAs in the BC microenvironment, proposing the possibility of anti-CAAs auxiliary therapy against BC development (Fig. 3). Further studies are required to investigate the CAA-associated strategy for better clinical outcomes of BC.

## Data Availability

Not Applicable.

## Abbreviations

BC: Breast cancer
CAAs: Cancer-associated adipocytes
TME: Tumor microenvironment
ECM: Extracellular matrix
miRNAs: microRNAs
WAT: White adipocytes
IL: Interleukin
CCL2: Chemokine ligand 2
CCL5: Chemokine ligand 5
EMT: Epithelial-mesenchymal transition
PKM2: Pyruvate kinase M2
FAK: Focal adhesion kinase
PLOD2: Lysyl hydroxylase 2
FAO: Fatty acid β-oxidation
TAMs: Tumor-associated macrophages
MT: Mammary tumor
TNF: Tumor necrosis factor
TGF: Transforming growth factor
HIF: Hypoxia-inducible factor
AMPK: AMP-activated protein kinase
mTOR: Mammalian target of rapamycin
mTORC1: mTOR complex 1
TSC2: Tuberous sclerosis 2
Raptor: Regulatory-associated protein of mTOR
TNBC: Triple-negative BC
IGF-1: Insulin-like growth factor 1
HGF: Hepatocyte growth factor
PDGF-BB: Platelet-derived growth factor-BB
IGFBP-2: Insulin-like growth factor binding protein 2
G-CSF: Granulocyte colony-stimulating factor
VEGF: Vascular endothelial growth factor
ATX: Autotaxin
LPA: Lysophosphatidic acid
HUVECs: Human umbilical vein endothelial cells
ADSCs: Adipose-derived stem cells
GSH: Glutathione
FFAs: Free fatty acids
ATP: Adenosine triphosphate
CAFs: Cancer-associated fibroblasts
ROS: Reactive oxygen species
TCA: Tricarboxylic acid
CAV1: Caveolin 1
MCT: Monocarboxylate transporter
ADFs: Adipocyte-derived fibroblasts
ATGL: Adipose triglyceride lipase
FABP5: Fatty acid-binding protein 5
3D: 3-dimensional
MET: Mesenchymal-to-epithelial transition
MMP: Matrix metalloproteinase
PAI-1: Plasminogen activator inhibitor type 1
YAP: Yes-associated protein
MSC: Mesenchymal stromal/stem cell
CLSs: Crown-like structures
MVP: Major vault protein
CSCs: Cancer stem cells
GDF15: Growth differentiation factor 15
IFN: Interferon
CAL: Cell-assisted lipotransfer
